# Medico-Legal Trial

**Published:** 1860-07-01

**Authors:** 


### MEDICO LEGAL TRIAL.

### DISPUTED WILL.—PLEA OF UNSOUND MIND.

### COURT OF PROBATE, MAY 18.

### Before Sir C. Cresswell and a Special Jury.

### WILLIAMS *v.* EVANS.

THE plaintiff, Anne Williams, propounded, as executrix, the will, dated the 5th of December, 1856, of Philip Taylor, late of Hirwaine, near Aberdare, in Glamorganshire, who died on the 23rd of July, 1859, at the age of 70. The defendant, Helen Eliza Evans, a niece of the testatrix, opposed probate on the grounds that the will was not duly executed, that the deceased was of unsound mind, and that it was obtained from him by the undue influence of the plaintiff.

Mr. Lush, Q.C., Dr. Spinks, and Mr. F. W. Lloyd were counsel for the plaintiff; Mr. Edwin James, Q.C., Mr. Serjeant Pigott, Dr. Swabey, Mr. Giffard, and Mr. Owen for the defendant.

The deceased began life as a draper's assistant, and afterwards carried on business for many years as a draper at Hirwaine. By this business, and by farming, and by some fortunate purchases of land which contained coal, he accumulated a fortune of about 15,000*l.* He was married, but had no children, and his wife died in 1848. A niece of his wife, Anne Edwards, the present plaintiff, lived with them until 1841. In that year she married David Evan Williams, who had been first Mr. Taylor's apprentice and then his partner, and who from 1837 had had the management of the business. Upon the marriage Mr. Taylor transferred the business to Williams for 1,700*l.*, of which he gave 200*l.* to Mrs. Williams, received 200*l.* from her father, and took a bond for the remaining 1,300*l.* from Williams, who paid him interest upon it. He gave up the premises upon which the business was carried on to the Williamses, and took up his own residence in an adjoining house. He had a niece, Helen Eliza Taylor, the daughter of an only brother, in Jamaica, and in 1841, at his request, she came over to England to live with him. She remained with him, keeping his house for him after his wife's death, until 1851, when she married Mr. Evans. She and her husband lived first at Cardiff, then at Manchester, and in October, 1855, went back to Hirwaine. The Williamses and the Evanses did not agree, and the deceased appeared at first to have sided with the Evanses, for in December, 1855, he gave the Williamses notice to quit the premises they occupied. The consequence of this notice was, that the Williamses removed to a third house adjoining the two occupied by the deceased and the Evanses. Between the death of his wife and 1855 the deceased made a number of wills. Their general effect was to divide his property between Mrs. Evans and Mrs. Williams, giving a slight advantage to Mrs. Evans, and to appoint them joint executrixes. In 1855 he made an alteration in this disposition. He gave the whole of his property to Mrs. Evans, appointing her sole executrix, subject to legacies of 500*l.* to Mrs. Williams and her five sisters, the daughters of Mr. Edwards, and these legacies were only to be payable conditionally. He had had a dispute with Mr. Edwards respecting the proportion to which he was entitled of a piece of land that they had purchased jointly; the land had turned out more valuable than had been anticipated in consequence of the discovery of coal, and the condition upon which the legacies were left was, that Mr. Edwards should transfer to him a sufficient quantity of the land to make up his share to 250 acres. His next will was made on the 10th of September, 1856. Mrs. Evans was appointed sole executrix and residuary legatee; 500*l.* was given to her sister and Mrs. Brown, 100*l.* to a friendly society for miners, 100*l.* to a former shopman, 6s. a week to an old servant, and 50*l.* a year to a Mrs. Moore. In June, 1856, Mrs. Evans had written to Mr. Frank James, the attorney of Mr. Taylor, directing him, as her uncle was getting very old and infirm and wished to transfer to her the management of his affairs, to prepare an instrument empowering her to act for him in all matters. She added that it would be necessary to make this instrument irrevocable, and that any document which he might subsequently sign should be void. A few days afterwards Mr. Taylor and Mrs. Evans went together to his office. He advised Mr. Taylor not to sign any such instrument, and drew up a short paper empowering Mrs. Evans to receive Mr. Taylor's rents as his agent, which Mr. Taylor signed. On the 2nd of December, 1856, he wrote to the secretary of the Vale of Neath Railway Company, asking him to transfer some shares which he held in that company to Mrs. Evans. The secretary sent him the forms which it was necessary for him to fill up before the transfer could be made. He received them on the 5th of December. On that day he went into Mr. Williams's shop, burst out crying, wished he was dead, showed the papers, said that the Evanses had been trying to get his property from him during his life, that they had treated him badly and he could stand it no longer, and asked to be allowed to live with Mr. and Mrs. Williams. They consented to receive him, and he said he should have come to them before, but he was afraid they had taken offence at having had notice to quit the premises they first occupied. He also said he had made a will disinheriting Mrs. Williams, and he wished to alter it immediately. Mrs. Williams asked him to take time for consideration, but he insisted, and a clergyman was sent for. The clergyman declined to interfere, and Mr. Frank James, his solicitor, was sent for. Mr. James and his clerk (Mr. Harris) arrived between eight and nine that same evening, saw the deceased alone, took his instructions for a will, drew it up, and it was duly executed and attested by Mr. James, Mr. Harris, and a chymist named Rees Thomas. By this will 70*l.* a-year in fee was left to Mrs. Evans, certain mortgage estates were vested in Mr. Williams, and Mrs. Williams was named residuary legatee and sole executrix. At the same time the instructions for the transfer of the Vale of Neath shares to Mrs. Evans, and the appointment of Mrs. Evans as agent for the receipt of rents, were revoked. He had left some deeds and papers in the house of the Evanses, who refused to give them up when they were demanded, and their detention gave rise to legal proceedings. In the beginning of 1857 the Evanses presented a petition to the Court of Chancery for a commission in lunacy to inquire into Mr. Taylor's state of mind. On the 13th of February, 1857, four medical men—Messrs. Dyke, Lucas, North, and White—had a long interview with him for the purpose of testing his capacity, and arrived at the conclusion that he was of perfectly sound mind, aud certified that opinion to the Lords Justices, before whom the petition was heard. Their lordships, thinking it desirable to have further evidence, commissioned Dr. Forbes Winslow to examine him. Dr. Winslow examined Mr. Taylor on five occasions —twice in London, and three times at Hirwaine—and stated the result in an elaborate report to their lordships. Dr. Winslow stated that Mr. Taylor was free from all kind of delusion, hallucination, and illusion; that he conversed about the Russian and Chinese wars and other topics of general interest; that he complained of the manner in which he had been treated by Mr. and Mrs. Evans, and accused them of having received money without accounting for it; that he said he had left their residence in consequence of having been asked to transfer some of his property to them, and had sought the protection of his other niece, Mrs. Williams; that he conversed about his property, and estimated its value at 15,000*l.*; that his knowledge of his pecuniary position appeared to be remarkably acute; and that the only part of his intellect which appeared to be defective was his memory, which occasionally flagged. Dr. Winslow said that, perceiving his irritation against Mr. and Mrs. Evans, he urged him to forget the past and make an equitable distribution of his property; to which he replied that he had not been well treated by them but would not forget their claim. In conclusion Dr. Winslow submitted that, if evidence similar to this were generally considered sufficient to warrant the protection of the Court of Chancery, no aged man in the kingdom with a memory somewhat impaired by age and bodily infirmity would be free from the suspicion of insanity and mental incapacity, and he reported that he was clearly of opinion that Mr. Taylor was a person of sound mind and capable of managing himself and his affairs. In consequence of this report their lordships, in April, 1857, dismissed the petition. In June, 1858, an application was made on the part of the Evanses for a *habeas corpus*, on the ground that the Williamses were detaining Mr. Taylor in their custody against his will, but that application also was refused, and with costs. The deceased died in July last of brain fever, having been confined to his bed for the previous 15 months.

In support of the plaintiff's case Dr. Forbes Winslow and the four medical men who had tested the testator's capacity in the course of the proceedings in Chancery in 1857 were examined, and adhered decidedly to the opinion they had then expressed. Mr. Edward Davies, a medical man at Merthyr Tydvil, had also examined him to test his capacity in December, 1856, and found him perfectly rational. This gentleman attended him subsequently, and said that in August, 1857, he had a slight attack of paralysis.

In addition to this medical evidence a great number of witnesses were called to speak to various acts of business done by the testator subsequent to the date of the will. Mr. Frank James and his partner, Mr. C. H. James, had had several consultations with him on the subject of the proceedings in Chancery and of the actions against the Evanses, and had taken his instructions, and had also transacted business for him connected with his mineral property at Hendrewin. On two occasions, in May and in September, 1857, Mr. C. H. James inquired whether he wished to make any alteration in the disposition of his property in favour of any of his other relations. He replied decidedly and positively that he wished matters to remain as they were. Mr. Joseph had seen him several times at the end of 1856 and the beginning of 1857 about taking a lease of the minerals at Hendrewin. He conducted the negociations himself and granted the leases. At the general election in 1857, he was canvassed for Mr. Vaughan, but said he had promised to vote for Messrs. Talbot and Vivian, and did vote for them.

A number of other witnesses were called to speak to various other smaller matters of business, such as the receipt and payment of money, in which he had acted for himself in 1856 and 1857, without showing any symptom of a decayed mind.

In the course of the case his lordship asked one of the witnesses what was the number of the inhabitants of Hirwaine, and how many of them knew the deceased.

The witness said Hirwaine had about 6,000 inhabitants, and most of them knew Mr. Taylor.

His lordship observed that he could now form some idea as to the probable duration of the cause.

The plaintiff's case was concluded, and Mr. Serjeant Pigott briefly opened the defendant's case.

The court then adjourned.

*May* 21.

This trial was resumed by the examination of Mr. William Davis, a surgeon, who had attended the deceased, and who said that he had an attack of paralysis in May, 1855, and that in June, 1856, he had become quite childish.

At the conclusion of this witness's examination, a conference took place between the learned counsel.

Mr. E. James then said that he and his learned friends found that it would be hopeless to attempt to establish the plea of undue influence on which the defendant relied, and he was therefore authorized to consent to a verdict for the plaintiff.

Mr. Lush said that the plaintiff was willing to hold out the olive branch by consenting that the costs should be paid out of the estate, in order that Mrs. Evans might enjoy the annuity of 70*l.* left her by the will.

His lordship observed that he hoped this arrangement would tend to the reconciliation of the parties. It was his opinion that the defendant had not the slightest chance of success, and he should have felt bound, however reluctantly, to have condemned her in costs, if the plaintiff had applied for them.

The jury expressed their concurrence in his lordship's opinion.

The verdict was accordingly entered for the plaintiff, and probate of the will of December, 1856, was granted; the costs, by consent, to be paid out of the estate.

